# Structural landscape of H3K27me3 recognition by protein domains and their potential for inhibition

**DOI:** 10.1016/j.jbc.2026.111344

**Published:** 2026-03-04

**Authors:** Ruben Rosas, Luisa F. Baracaldo-Lancheros, Emily C. Dykhuizen, Catherine A. Musselman

**Affiliations:** 1 Department of Biochemistry and Molecular Genetics, University of Colorado School of Medicine, Aurora, Colorado, USA; 2 Department of Medicinal Chemistry and Molecular Pharmacology, Purdue University, West Lafayette, Indiana, USA

**Keywords:** chromatin, nucleosome, H3K27me3, chromodomain, BAH domain, Tudor domain, WD40 domain, inhibitor

## Abstract

A fraction of the eukaryotic genome is transcriptionally silenced in the form of facultative heterochromatin, characterized by the histone H3 lysine 27 tri-methyl (H3K27me3) modification. The cell-specific and dynamic nature of H3K27me3-marked chromatin is centrally regulated by the catalytic function of the polycomb repressive complex 2 (PRC2) that deposits it; however, the mark can also be removed to activate transcription by the demethylases UTX and JMJD3. An important regulatory mechanism of facultative heterochromatin is the molecular recognition of the H3K27me3 modification by a group of small globular proteins termed readers. Across multiple organisms, the readers of H3K27me3 that have been structurally characterized bound to H3 peptides are restricted to the chromodomain, BAH, Tudor, and the WD40 EED. Here, we review the structural diversity of the protein domains that bind to H3K27me3 and highlight the different binding preferences beyond the recognition of the K27me3 moiety. Furthermore, we note recent findings that suggest the nucleosome structure can enhance the specificity of readers for H3K27me3, adding a new layer of regulation. Finally, we discuss the prevalence of misregulation of H3K27me3 and its cognate proteins in human diseases, and the potential of the latter for therapeutic intervention. Remarkably, almost all the H3K27me3-related proteins are found misregulated in malignances that affect the brain and the nervous system, along with a strong prevalence in cancers of other tissues. Pharmacological efforts to target these pathways include peptide-based inhibitors and small molecules that can block recognition of H3K27me3 by allosteric, complex-disruptive, or degradation-inducing mechanisms of inhibition.

## H3K27me3 marks facultative heterochromatin

**Figure 1:**
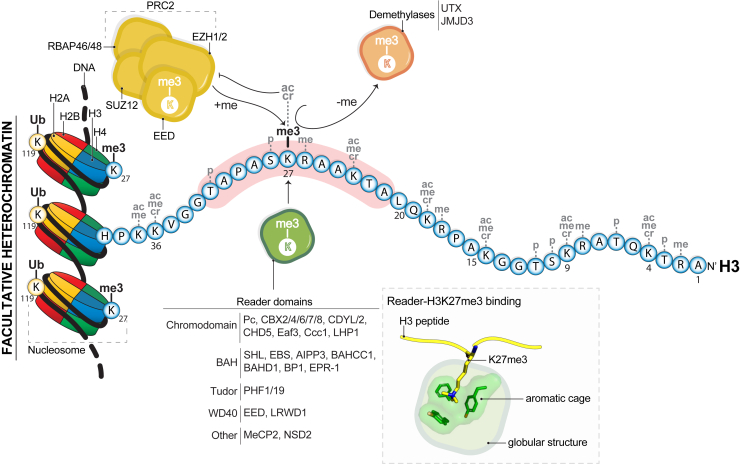
**H3K27me3 is a hallmark of the transcriptionally silenced facultative heterochromatin**. H3K27me3 is deposited on nucleosomal H3 N-terminal tails (residues 1–39) by PRC2 where the “Kme3” moiety is recognized by a plethora of reader domains including EED in PRC2 and lysine demethylases that in addition specifically interact with residues around K27 (*red shadow*). Besides K27me3, the side chain of some amino acids on the H3 tail can be modified with other posttranslational modifications including various levels of methylation (me), acetylation (ac), crotonylation (cr), and phosphorylation (p) (5). The ubiquitylation (Ub) of K119 of nucleosomal histone H2A is catalyzed by the PRC1 complex and is also a mark of facultative heterochromatin. Readers of H3K27me3 recognize the methyllysine through an aromatic cage that is typically composed of 3 or 4 aromatic residues within the globular structure of the domain. EED, embryonic ectoderm development; H3K27me3, tri-methyl lysine 27; PRC2, polycomb repressive complex 2.

The repeating unit of chromatin is characterized by approximately 147 base pairs (bp) of DNA wrapped around two copies of the histone proteins H3, H4, H2A, and H2B in a globular arrangement known as the nucleosome (Fig. 1). Nucleosomes not only provide protection and organization to the eukaryotic genome but also regulate virtually any cellular function that requires reading of the genomic information encoded in the DNA including transcription, DNA replication, and DNA repair. One regulatory mechanism of chromatin structure is the incorporation of posttranslational modifications (PTMs) onto the histones. The majority of these PTMs are found on the unstructured N terminal tails that protrude out of the nucleosome core.

Notably, the N terminal tail of histone H3 is the longest histone tail and serves as a signaling hub acquiring many PTMs including tri-methyl (me3) groups on lysines (Fig. 1). This PTM makes the lysine side change more hydrophobic and alters the hydrogen bonding capacity. The enrichment of Kme3 at specific chromatin loci is correlated with the transcriptional state of the underlying genes depending on the exact lysine residue that harbors the modification. Histone H3 tri-methyl lysine 27 (H3K27me3) is usually found on transcriptionally inactive genes (1) within a higher order chromatin structure termed facultative heterochromatin (Fig. 1) (2, 3). This type of structure is characterized by a condensed aggregation of nucleosomes and chromatin-binding proteins that are tightly packed to block binding of transcription factors to the DNA. Facultative heterochromatin is dynamic, cell-linage specific, and its organization is triggered by developmental cues. The incorporation of H3K27me3 can establish facultative heterochromatin *de novo* or promote its maintenance whereby H3K27me3 is required for chromatin compaction.

In mammals, the trimethylation of H3K27 is exclusively catalyzed by the protein complex polycomb repressive complex 2 (PRC2) (reviewed in (4)). Although several proteins can associate with PRC2 to form variants of the complex, granting specialized functions, there are four core subunits of PRC2: suppressor of zeste 12 (SUZ12), embryonic ectoderm development (EED), enhancer of zeste homolog 1/2 (EZH1/2), and retinoblastoma-binding protein 48/46 (RBAP48/46, a.k.a RBBP4/7). EZH1/2 is the catalytic subunit that carries the methyltransferase activity on unmodified H3K27 (6) while the EED, SUZ12 and RBAP48/46 subunits act as scaffolding structures and activators of the EZH1/2 enzymatic activity through allosteric mechanisms (Fig. 1). The two homologues of EZH are mutually exclusive within PRC2, where PRC2-EZH2 has a more potent methyltransferase activity than PRC2-EZH1 (6). Removal of H3K27me3 coincides with gene activation, which can be achieved by H3K27me3-specific demethylases (Fig. 1) (see “Lysine Demethylases” section below) or by dilution of the mark through cell division (6, 7, 8, 9, 10).

A substantial part of H3K27me3-mediated biological function is regulated through the nonenzymatic recognition of H3K27me3 by histone H3 reader domains (Fig. 1). Importantly, nearly all of these proteins are implicated in human disease and are strong therapeutic targets. In the following sections, we will discuss the unique protein and structural features of the reader domains that allow for unique recognition of H3K27me3 and the current pharmacological strategies to selectively target H3K27me3-binding proteins.

## Signaling function: how is H3K27me3 read?

Many protein domains specifically bind to H3K27me3, and the mechanisms underlying these interactions have been characterized using short peptides corresponding to the histone H3 tail. These structural studies have revealed that the recognition of H3K27me3 is not exclusive to a single type of protein fold or domain but rather that H3K27me3 binding is permissive to structural variations. A general feature, however, is the presence of an aromatic cage or pocket that directly coordinates the K27me3 modified residue (Fig. 1). The cage is typically made of 3 or 4 aromatic residues that together create a shallow and planar hydrophobic configuration that is solvent exposed and on the surface of a globular tertiary protein structure where it can perfectly accommodate the side chain of the methylated lysine. The interaction between the aromatic cage and the methyl-lysine is stabilized predominantly by cation-π interactions between the aromatic rings and the positive charge of the lysine amino group. Given that the N terminal tail of histone H3 can be trimethylated at other lysine residues, the specificity of H3K27me3 readers comes from protein surfaces beyond the aromatic cage that engage or are compatible with the H3 residues around K27 (Fig. 1). Notably, when investigated *in vitro* using histone H3 tail peptides a low degree of specificity is commonly observed between reader domains and the H3K27me3 *versus* H3K9me3 marks. This similarity in substrate preference is largely explained by the “ARKS” sequence homology surrounding K9 and K27 (Fig. 1). However, recent experiments *in vitro* indicate that histone reader domains may gain specificity between H3K27me3 and H3K9me3 when the mark is in the context of the nucleosome (11), suggesting that the nucleosome structure can regulate the accessibility of the reader domains for histone PTMs (see section “H3K27me3 binding in chromatin relevant context”). Below, we highlight and discuss the structural details of known interactions between protein reader domains and the H3K27me3 mark (Table 1).

**Table 1:** List of proteins known to bind to H3K27me3

Type of epigenetic regulator	Domain	Protein	Species	H3K27me3 inhibitor?
Reader	CHROMODOMAIN	Pc	*Drosophila melanogaster*	-
		CBX2	Mammals	yes
		CBX4	Mammals	yes
		CBX6	Mammals	yes
		CBX7	Mammals	yes
		CBX8	Mammals	yes
		CDYL	Mammals	yes
		CDYL2	Mammals	yes
		CHD5	Mammals	no
		Eaf3	*Magnaporthe oryzae*	-
		Ccc1	*Cryptococcus neoformans*	-
		LHP1	*Arabidopsis thaliana*	-
	BAH	SHL	Plants	-
		EBS	Plants	-
		AIPP3	Plants	-
		BAHCC1	Mammals	no
		BAHD1	Mammals	no
		BP1	*Fusarium graminearum*	-
		EPR-1	*Neurospora crassa*	-
	TUDOR	PHF1	Mammals	Yes
		PHF19	Mammals	Yes
	WD40	EED	Eukaryotes	Yes
		LRWD1	Vertebrates	No
	Other	MeCP2	Vertebrates	No
		NSD2	Metazoans	No
Eraser	JmjC	UTX	Metazoans	Yes
		JMJD3	Metazoans	Yes

JmjC, Jumonji C.

### Chromodomain

Chromodomains constitute small globular protein domains that encompass about 40 to 60 amino acids and typically bind to methylated lysines, including H3K27me3, with mid-to-high micromolar binding affinities. Beyond histone recognition, chromodomains have been found to also associate with nucleic acids and other proteins, therefore exhibiting a wide regulatory repertoire within cellular processes.

**Figure 2:**
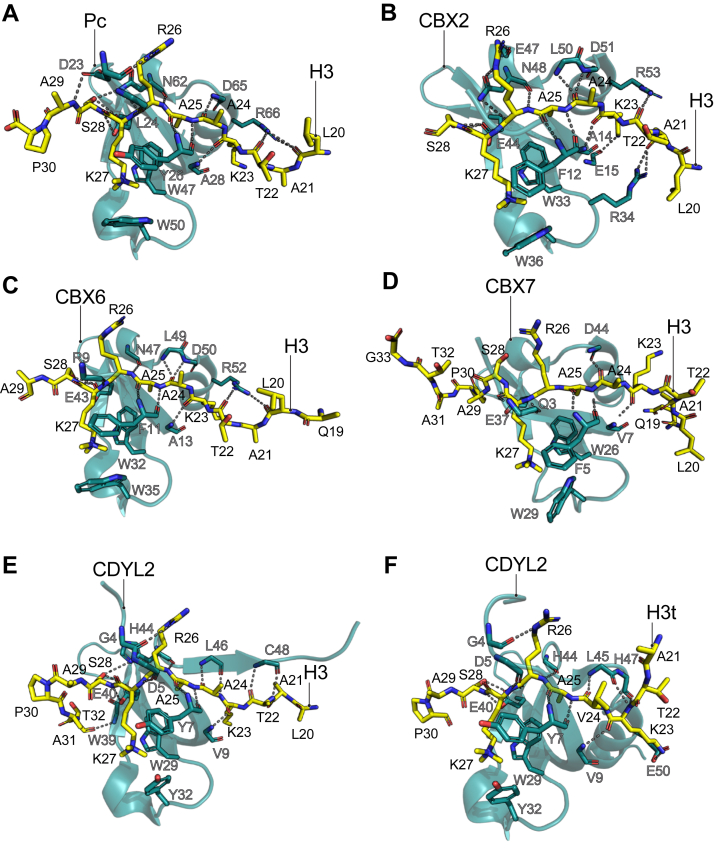
**Chromodomains recognize H3K27me3 through a conserved aromatic cage and extensive hydrogen bonding with residues upstream of K27 in H3**. Solved structures of H3K27me3 peptides in complex with chromodomains from *D*. *melanogaster* Pc (*A*, obtained *via* X-ray diffraction with PDB ID: 1PBFB), *Homo sapiens* CBX2 (*B*, obtained *via* X-ray diffraction with PDB ID: 3H91), *H*. *sapiens* CBX6 (*C*, obtained *via* X-ray diffraction with PDB ID:3I90), *H*. *sapiens* CBX7 (*D*, obtained *via* NMR showing model 1 from PDB ID: 2L1B), and *H*. *sapiens* CDYL2 (*E*, PDB ID: 6V3N and *F*, PDB ID: 6V2H; both obtained *via* X-ray diffraction). The H3 peptides are represented in a stick model with their amino acids labeled in *black letters*. The chromodomains are shown in a cartoon representation with sections of some residues that participate in the binding to the H3 peptide shown in stick models and labeled in *gray* letters. Hydrogen bonds between the H3 peptide and the chromodomain are represented as *dotted lines*. H3t corresponds to a peptide from the testis-specific histone H3 variant. CBX, chromobox; CDYL2, chromodomain on the Y like protein 2; H3K27me3, Histone H3 tri-methyl lysine 27; PDB, Protein Data Bank.

The first protein known to specifically interact with H3K27me3 was the PRC1 subunit Polycomb (Pc) from *Drosophila melanogaster*. Using pull-downs and recombinantly produced Pc, it was determined that this protein preferentially binds to H3K27me3 *in vitro*; a function that was largely attributed to the chromodomain located at the N terminus of the Pc polypeptide (12, 13). Similar results were also observed when using a mammalian homolog of Pc (14). Two independent groups simultaneously solved the crystal structure of the chromodomain of *D*. *melanogaster* Pc in complex with an H3K27me3 peptide (15, 16) (Fig. 2*A*). The high-resolution set of structures converged at revealing a canonical chromodomain tertiary structure composed of three β-strands that are arranged into a twisted antiparallel β-sheet and packed against a short C-terminal α-helix (Fig. 2*A*). The aromatic cage of Pc that binds to the K27me3 is formed by two Trp and one Tyr residue (Fig. 2*A*). Although longer peptides were employed, only H3 residues 20 to 30 showed interpretable electron density in at least one of the crystal structures, suggesting that H3 residues beyond this sequence are conformationally heterogenous and are unlikely to contribute to binding the chromodomain (15). Importantly, the H3K27me3 peptide appears extended in a β-sheet-like conformation that is stabilized by hydrogen bonding of the backbone of H3 residues 23 to 26 with several residues in Pc (Fig. 2*A*) (16). H3L20, T22, and S28 also formed hydrogen bonds with Pc in the crystal structure (Fig. 2*A*). Finally, H3 A25 was bound to Pc by hydrophobic interactions in a shallow surface in Pc (15, 16). Together, the *D*. *melanogaster* Pc chromodomain structure demonstrated recognition of H3K27me3 through an extended peptide binding groove that not only accommodates the K27me3 but that is highly compatible with the H3 residues 20 to 28.

Mammalian homologs of Pc are chromobox (CBX) 2, 4, 6, 7, and 8, which are known to form mutually exclusive PRC1 complexes (17). The chromodomain within the CBX polypeptide has been proposed to be able to recruit the PRC1 complex to H3K27me3 chromatin sites in a manner similar to Pc in *D*. *melanogaster*. The structure of the chromodomains from human CBX2/6/7, and mouse CBX7 in complex with an H3K27me3 peptide have been solved using either X-ray crystallography or nuclear magnetic resonance (NMR) (Fig. 2, *B*–*D*) (18, 19). As expected from the 78 to 82% sequence similarity between the chromodomain of *D*. *melanogaster* Pc and the chromodomain in human and mouse CBXs (20), the mammalian CBX structures showed identical tertiary structure to the Pc chromodomain. The methyl-lysine binding aromatic pocket is formed by two Trp and one Phe instead of a Tyr as compared to Pc and also demonstrates an extensive hydrogen binding network between each CBX chromodomain and the H3 peptide. Moreover, as in the Pc structure, A25 of the H3 peptide is also fitted into a shallow hydrophobic surface within the CBX chromodomains (Fig. 2, *A*–*D*) (18). Intriguingly, despite the striking similarities between the mammalian and fly chromodomains, the CBX chromodomains have a 20 to 100-fold decrease in binding affinity to H3K27me3 peptides when compared to the fly Pc ((15), 18). This difference in binding affinity has been largely attributed to a less acidic character of the mammalian CBX chromodomain homologs that makes them less compatible with the basic H3 peptide (18).

Another chromodomain-containing protein that has been structurally investigated for the recognition of H3K27me3 is the chromodomain on the Y like protein 2 (CDYL2). CDYL2 belongs to a small sequence-related family of mammalian proteins that are predominantly expressed in testis and are important for normal spermatogenesis and brain development (21). CDY proteins contain a conserved N-terminal chromodomain that can bind to histone methyl-lysine peptides and a C-terminal crotonase-like catalytic domain that converts available crotonyl-CoA to β-hydroxybutyryl-CoA, which in turn negatively regulates histone lysine crotonylation and suppresses transcription (21, 22). In addition, CDYL recruits PRC2 to discrete genomic loci through direct interactions with the EZH2 subunit to deposit H3K27me3 (23). *In vitro*, the chromodomain of CDYL2 binds to H3K27me3 peptides with a modest preference for the testis-specific histone H3 peptide (H3tK27me3) which has a valine residue instead of an alanine at position 24 of H3 (21). Using X-ray crystallography, the structure of the human CDYL2 chromodomain was solved in complex with both canonical and testis-specific H3K27 peptides (Fig. 2, *E* and *F*). The two structures showed an overall identical configuration on the β-sheet arrangement of the globular CDYL2 chromodomain as well as on the H3 peptide that resembles that of the *D*. *melanogaster* Pc chromodomain-H3K27me3 structure (Fig. 2*A*). However, in the CDYL2 chromodomain structure in complex with the canonical H3K27me3 peptide, the C-terminal α-helix of the chromodomain is found displaced from the chromodomain β-sheet core, presumably due to a crystallographic artifact (21). The CDYL2 chromodomain structures revealed that the preference for the H3tK27me3 peptide over the canonical H3K27me3 is largely due to Val24 in the H3t peptide that allows for an extensive hydrophobic interaction between the peptide and the chromodomain as compared to the canonical H3K27me3 (21).

In humans, another chromodomain-containing protein reported to have H3K27me3 binding function is the chromodomain-helicase-DNA-binding protein 5 (CHD5) with this interaction being required for neuronal differentiation (24). In addition, the proteins ESA1-associated factor 3 (Eaf3) in *Magnaporthe oryzae*, chromodomain-containing subunit 1 (Ccc1) in *Cryptococcus neoformans*, and like heterochromatin protein 1 (LHP1) in *Arabidopsis thaliana*, also bind to H3K27me3 through their chromodomains to maintain gene silencing through a positive feedback loop mechanism with PRC2 (25, 26, 27, 28). The structural basis for binding of these has yet to be elucidated.

Finally, the chromodomains that preferentially bind to the H3K9me3 mark that also contain the “ARKS” motif (Fig. 1) show slight differences when compared to the H3K27me3-chromodomains. In proteins such as HP1 from *Drosophila* and CBX1/3/5 in mammals the aromatic cage is characteristic of a narrower and deeper cavity where the rest of the H3K9me3 peptide is fitted in or surrounded by acidic residues that increase retention of the basic H3 peptide to the domain (18, 29). These structural features create the conditions for a more stable binding interface between H3K9me3 chromodomains in contrast to a more dynamic and sequence permissive binding mode in H3K27me3-binding chromodomains.

### Bromo-adjacent homology (BAH) domain

Bromo-adjacent homology (BAH) domains are a conserved family of protein motifs containing 120 to 140 amino acids predominantly forming a core of β-sheets. These domains are mainly found in nuclear proteins and can recognize a variety of substrates including methylated and unmethylated histone proteins, the nucleosome core structure, and other proteins.

The initial structural characterization of how BAH domains bind to H3K27me3 comes from investigations of *A*. *thaliana* and *Populus trichocarpa* organisms. Using methylated histone peptide pull-downs, the plant BAH-containing proteins short life (SHL), Early bolting in short day (EBS), and ASI1-immunoprecipitated protein 3 (AIPP3, a.k.a. BDT1) were identified as H3K27me3 binders. This function was also shown to be essential to repress genes that prevent early flowering during plant development (30, 31, 32, 33).

**Figure 3:**
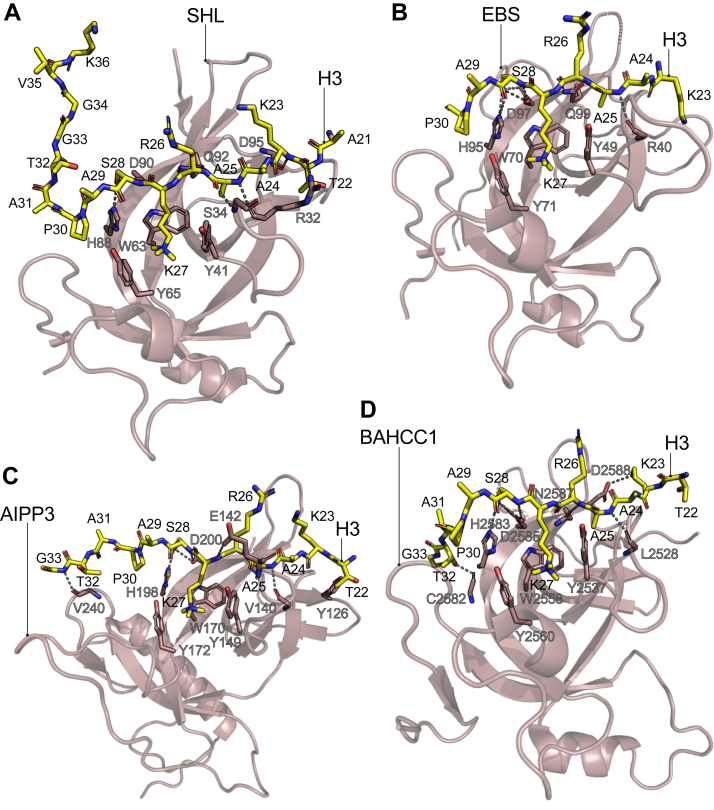
**H3P30 forms CH-π interactions with a histidine in the BAH domains to selectively recognize H3K27me3**. X-ray crystal structures of H3K27me3 peptides in complex with BAH domains from *P*. *trichocarpa* SHL (*A*, PDB ID: 5ZNR), *A*. *thaliana* EBS (*B*, PDB ID: 5Z8L), *A*. *thaliana* AIPP3 (*C*, PDB ID: 7CCE), and *Mus musculus* BAHCC1 (*D*, PDB ID: 6VIL). The H3 peptides are represented in a stick model with their amino acids labeled in *black* letters. The BAH domains are shown in a cartoon representation with sections of some residues that participate in the binding to the H3 peptide shown in stick models and labeled in *gray* letters. Hydrogen bonds between the H3 peptide and the BAH domain are represented as *dotted* lines. BAHCC1, bromo-adjacent homology domain and coiled-coil containing 1; EBS, bolting in short day; H3K27me3, Histone H3 tri-methyl lysine 27; PDB, Protein Data Bank; SHL, short life.

X-ray crystal structures of these plant BAH domains in complex with H3K27me3 peptides showed a conserved distorted β-barrel tertiary structure. The H3K27me3 peptide binds to a negatively charged cavity on the BAH surface, with the methyl-lysine in an aromatic cage formed by a Trp at the back and a Tyr on either side (Fig. 3, *A*–*C*). In addition, several hydrophobic contacts and hydrogen bonds help anchor the H3 peptide to the BAH structure. However, the H3 peptide does not acquire a β-sheet conformation as with chromodomains. Remarkably, the BAH structures revealed that most of the selectivity for the H3K27me3 peptide comes from the recognition of proline 30 on the histone H3 peptide. In all three BAH-H3K27me3 structures, the prolyl ring of Pro30 on the H3 peptide is found aligned parallel and forming CH-π and stacking interactions with an imidazole ring of a conserved His residue on the BAH (Fig. 3, *A*–*C*). This alignment is further favored by hydrogen bonding of the side chain of Ser28 on H3 with the His residue along with the side chain of a neighboring Asp amino acid in the BAH (Fig. 3, *A*–*C*). Mutation of these conserved BAH His and Asp residues involve in the recognition of H3 Pro30 reduced the binding affinity toward the H3K27me3 peptide similarly to a mutation in the methyl-lysine aromatic cage residues (31, 32, 33).

In mammals, orthologs of the plant BAH domains that bind to H3K27me3 have been identified. The human proteins bromo-adjacent homology domain and coiled-coil containing 1 (BAHCC1) and bromo-adjacent homology domain-containing protein 1 (BAHD1) harbor a BAH domain that binds to H3K27me3 to maintain gene repression of specific chromatin loci (34, 35, 36, 37). Unlike the chromodomain of CBX proteins that recruit PRC1, the human BAHCC1 and BAHD1 BAH domains maintain facultative heterochromatin by recruiting and binding to histone deacetylases to maintain low acetylation levels at PRC2-target genes (35, 36). The X-ray crystal structure of the mouse BAHCC1 BAH domain in complex with an H3K27me3 peptide showed an identical mode of binding to that of the plant BAH domains, with an aromatic cage formed by Trp and Tyr residues and H3P30 specifically recognized by conserved His and Asp residues in the BAH domain (Fig. 3*D*) (35, 36).

Other BAH-containing proteins that read H3K27me3 have been reported in fungi. In *Fusarium graminearum*, the protein bromo-Adjacent homology plant homeodomain containing protein 1 (BP1) binds to H3K27me3 chromatin regions through a BAH domain to corepress gene transcription, where part of the silencing mechanism involves the direct interaction of BP1 with the PRC2 component SUZ12 (38, 39). Finally, in *Neurospora crassa* the protein effector of polycomb repression 1 (EPR-1) contains a BAH domain that contributes to the colocalization of EPR-1 to transcriptionally silence genes at H3K27me3 chromatin regions through an unknown molecular mechanism (40).

Interestingly, an inspection of the structure of the BAH domain from human TNRC18 in complex with an H3K9me3 peptide reveals a mechanism of selectivity between the H3K27me3 and H3K9me3 binding BAH domains that involves H3 residues outside the “ARKS” motif (Fig. 1). Although S28 and P30 in the H3K27me3 substrate are essential for binding to H3K27me3-BAHs (Fig. 3), the side chain of T6 in H3K9me3 binds specifically to the BAH of TNRC18 through hydrogen bonding and van der Waals interactions (41). Therefore, these structural differences in H3 substrate recognition promote a high degree of specificity between H3K9me3 and H3K27me3 reader BAH domains.

### Tudor domain

Tudor domains form small globular tertiary structures composed of about 60 residues that preferentially associate with proteins, including histones, that are methylated at lysine or arginine residues. The functional mechanism of Tudor domains can involve individual domain units, tandem Tudor domains, or hybrid domains, which can alter binding specificity (42). The PRC2-interacting proteins PHF1 and PHF19 contain a Tudor domain that has been largely associated with the recognition of the H3K36me3 mark to recruit PRC2 to transcriptionally active chromatin regions (6, 43). In addition to the histone binding function, the Tudor domain of PHF1 can also interact with DNA, which synergistically with H3K36me3 binding may stabilize the nucleosome in a more open conformation *in vitro* (44).

**Figure 4:**
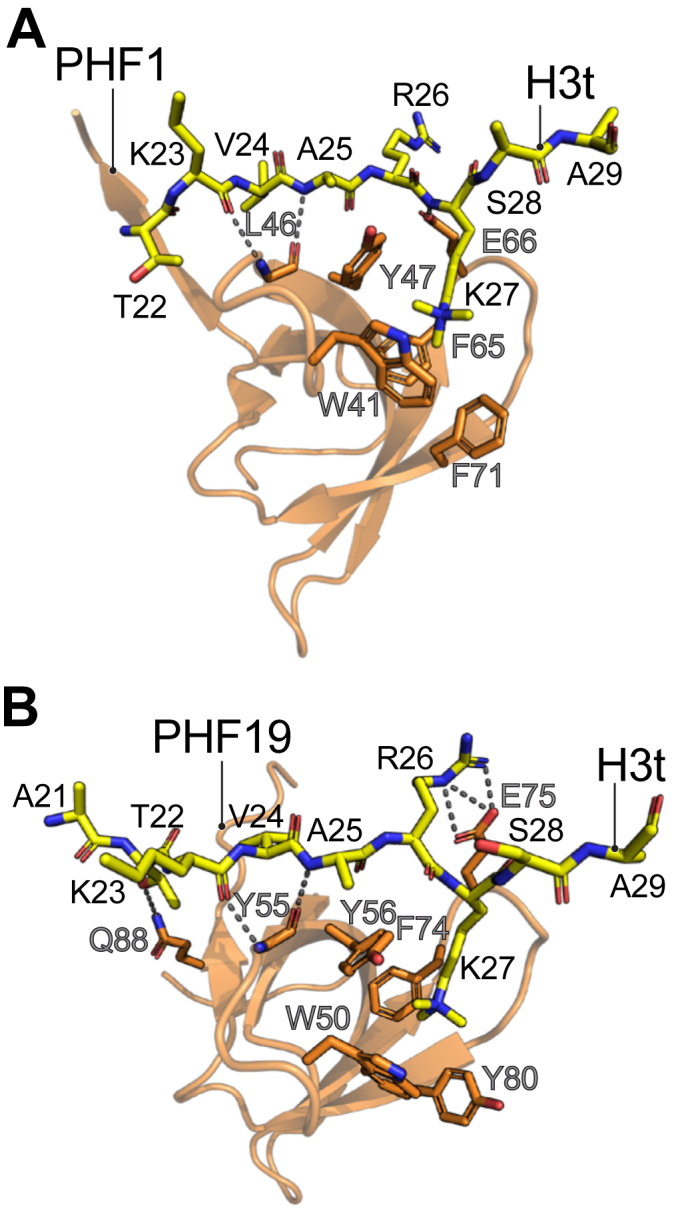
**The A24V substitution present in the testis-specific histone H3 variant (H3t) is preferentially recognized by the PHF1/19 Tudor domains**. X-ray crystal structures of H3tK27me3 peptides in complex with Tudor domains from *Homo sapiens* PHF1 (*A*, PDB ID: 6WAT), and *H*. *sapiens* PHF19 (*B*, PDB ID: 6WAU). The H3t peptides are represented in a *stick* model with their amino acids labeled in *black* letters. The Tudor domains are shown in a *cartoon* representation with sections of some residues that participate in the binding to the H3t peptide shown in stick models and labeled in *gray* letters. Hydrogen bonds between the H3t peptide and the Tudor domain are represented as *dotted lines*. PDB, Protein Data Bank

Besides binding to H3K36me3 chromatin, PHF1 and PHF19 also colocalize at H3K27me3 chromatin through their Tudor domain (43, 45, 46, 47). Similarly to the chromodomain of CDYL2, *in vitro* binding assays using histone peptides and recombinantly purified Tudor domains further indicated that PHF1 and PHF19 preferentially bind to the testis-specific H3tK27me3 over canonical H3K27me3 (45, 46). The X-ray crystal structures of PHF1 and PHF19 Tudor domains in complex with an H3tK27me3 peptide revealed an unperturbed canonical folding of the five-stranded antiparallel β-barrel Tudor domain (Fig. 4, *A* and *B*). The methyl-lysine binding aromatic cage of the Tudor domains is wide and composed of four aromatic residues that differ only at the bottom position with Phe in PHF1 and Tyr in PHF19 (Fig. 4, *A* and *B*). In addition to the aromatic residues, the cage cavity is also formed by an Asp and a Ser residue that contribute to the methyl-lysine recognition with polar interactions (46). The H3tK27me3 peptide is stabilized on the Tudor domain surface by hydrophobic interactions and hydrogen bonding. A particular region on the Tudor globular surface is a leucyl patch that is mainly formed by a cluster of Leu resides and makes extensive hydrophobic interactions with Val24 on the H3tK27me3 peptide (Fig. 4, *A* and *B*). These hydrophobic interactions are favored by the isopropyl side chain in Val24 in H3tK27me3 as opposed to Ala24 in the canonical H3K27me3 peptide and explain the binding preference of the PHF1 and PHF19 Tudor domains for the H3t peptide. In agreement, mutations to the residues in the leucyl patch reduce the binding affinity towards the H3tK27me3 peptide (46).

### WD40–EED subunit

**Figure 5:**
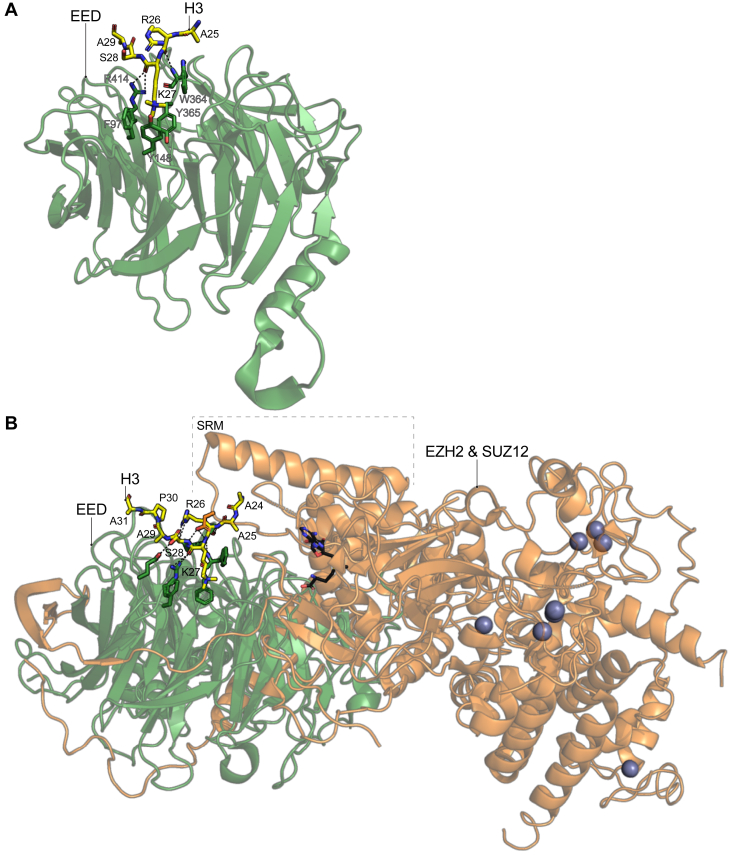
**PRC2 recognizes its own catalytic product mainly through the aromatic cage in the EED subunit; however, the SRM in EZH2 also binds to H3 providing specificity for H3K27me3**. *A*, X-ray crystal structure of the *Homo sapiens* EED subunit in complex with an H3K27me3 peptide (PDB ID: 3IIW). *B*, X-ray crystal structure of a minimally functional PRC2 complex from *Chaetomium thermophilum* bound to an H3K27me3 peptide (PDB ID: 5KKL), with *silver spheres* representing Zn^2+^ ions coordinated by PRC2 and in a *black* stick model is the EZH2 cofactor S-adenosyl methionine (SAM) for methyl transferase activity. The H3 peptides are represented in a stick model with their amino acids labeled in *black* letters. The PRC2 subunits are shown in a cartoon representation with sections of some residues that participate in the binding to the H3 peptide shown in stick models and labeled in *gray* letters for the human EED in (*A*). Hydrogen bonds between the H3 peptide and PRC2 subunits are represented as *dotted lines*. EED, embryonic ectoderm development; H3K27me3, Histone H3 tri-methyl lysine 27; PDB, Protein Data Bank; PRC2, polycomb repressive complex 2; SRM, stimulation-responsive motif.

The EED core subunit of PRC2 binds to H3K27me3, and this interaction is essential in the read-write mechanism of PRC2 to spread H3K27me3 across the genome. The observation that all the core subunits of the PRC2 complex are required for colocalization with H3K27me3 regions during DNA replication (48) partially inspired the search for a protein surface within PRC2 that could “read” this histone mark. Conveniently, the previously solved X-ray crystal structure of the mouse EED subunit displayed a cavity within the top surface that resembled that of an aromatic cage that could accommodate a methyl-lysine substrate (49). Subsequent *in vitro* studies with recombinantly produced human EED and histone peptides confirmed that EED can bind to H3K27me3 and provided high-resolution crystal structures of the interaction (Fig. 5*A*) (50, 51).

Unlike the subdomains described above, the majority of the EED polypeptide is required to properly achieve the globular structure that recognizes H3K27me3 (Fig. 5*A*). The tertiary structure of the EED subunit belongs to the WD40 repeat family of proteins, where repetitive sequence motifs of Gly-His and Trp-Asp fold into small β-sheets made of four antiparallel β-strands that together are assembled into a circularized β-propeller globular structure (Fig. 5*A*) (52). The X-ray crystal structures of the human EED in complex with an H3K27me3 peptide showed that the methyl-lysine fits into the aromatic cage formed by two Tyr, a Phe and a Trp residue on the top surface of the globular protein (Fig. 5*A*) (50, 51). In contrast to other readers discussed, few additional contacts were observed with only hydrogen bonds between the backbone of R26 and K27 in H3 with the EED protein (Fig. 5*A*).

The structure of a minimally functional PRC2 complex from the fungus *Chaetomium thermophilum* has been solved in complex with an H3K27me3 peptide bound to its EED subunit (Fig. 5*B*) (53). Consistent with a functional conservation, the X-ray crystal structure revealed an identical mode of binding of the *C*. *thermophilum* EED to the H3K27me3 peptide when compared to the human ortholog (Fig. 5, *A* and *B*). However, the EZH2 subunit, included in the crystallized *C*. *thermophilum* PRC2 complex, made additional contacts with the H3K27me3 peptide through its stimulation-responsive motif (SRM) which “sandwiches” the H3K27me3 peptide between it and EED (Fig. 5*B*). Importantly, the SRM motif acquires an alpha-helical conformation only in the presence of the H3K27me3 peptide with no interpretable electron density observed without the H3K27me3 ligand (53). This structural rearrangement is part of the molecular mechanism for allosteric stimulation of PRC2 activity upon binding H3K27me3 (53). Although the recognition of H3K27me3 by PRC2 is largely attributed to the EED subunit, the narrow cavity formed between both the EED and EZH2-SRM is highly compatible with the sequences around H3K27, providing an explanation as to why a marked preference toward H3K27me3 substrates is mainly observed when using the entire PRC2 complex and not the individual EED subunit (50, 53).

Furthermore, cryogenic electron microscopy (cryo-EM) reconstitutions of human PRC2 with dinucleosome chromatin substrates revealed that a single PRC2 complex can engage with an H3K27me3 modified nucleosome through the EED subunit while the catalytic EZH2 subunit is mostly positioned toward the adjacent unmodified nucleosome (54). Interestingly, this PRC2 arrangement is permissive to various linker DNA lengths, suggesting that PRC2 can act on various chromatin landscapes. Together, these structural studies have elucidated some of the chromatin substrate requirements for the H3K27me3 read-write mechanism of PRC2 between neighboring nucleosomes (54).

At least one other WD40-containing protein has been identified to interact with H3K27me3. Using human cell lysates and mass spectrometry coupled with pull-downs using either methylated histone peptides or *in vitro* reconstituted H3K27me3-nucleosomes, the leucine-rich repeat and WD repeat-containing protein 1 (LRWD1, a.k.a. ORCA) was identified as a reader of the H3K27me3 repressive mark (55, 56). LRWD1 associates with the origin of recognition complex (ORC) and contains a WD40 domain that is likely to recognize H3K27me3 in chromatin; however, the details of the LRWD1-H3K27me3 interaction are yet to be determined (55, 56).

### Other reader domains

Besides the structural motifs discussed above, other proteins that have been reported to function as H3K27me3 readers in humans are the methyl-CpG binding protein 2 (MeCP2) and the nuclear SET domain-containing protein 2 (NSD2, a.k.a. MMSET and WHSC1).

MeCP2 can act as both a transcriptional activator or repressor, and it is important for mature nerve cells being found highly expressed in neurons (57, 58). The recruitment of MeCP2 to the genome has been largely attributed to its N-terminal methyl-CpG-binding domain (MBD) that can specifically bind to various, methylated and unmethylated, DNA motifs through the recognition of the hydration of the major groove of the DNA (59). However, *in vitro* pull-downs and immunoprecipitation assays with reconstituted nucleosomes indicate a preference of MeCP2 for H3K27me3 chromatin (55, 60). This preference was recapitulated only when using a purified MeCP2 construct that contains the MBD region and a preformed complex of MeCP2 MBD bound to H3K27me3 nucleosomes was exclusively displaced by an H3K27me3 peptide in competition assays (60). Furthermore, many of the functional impacts of MeCP2 on gene regulation correlated with MeCP2 colocalizing at H3K27me3 chromatin regions (60). These results agree with an H3K27me3 reader role for the MBD of MeCP2; however, the molecular details of MBD-H3K27me3 interaction are yet to be determined.

In humans, NSD2 is an H3K36 specific lysine methyltransferase that broadly regulates genes involved in development and DNA repair (61). NSD2 harbors a zinc finger cassette composed of a canonical plant homeodomain (PHD) and a C5HCH zinc coordinating motif that together are referred to as PHDvC5HCH (62). Recently, using a combination of structural biology techniques the PHDvC5HCH of NSD2 was characterized as an H3K27me3 reader domain that utilizes two protein surfaces to bind to long histone H3 sections (residues 1–30) that contain K27me3 (62). The PHD region binds to the N terminal portion of the H3 recognizing the unmodified K4 while the K27me3 is specifically recognized by an aromatic cage located within the C5HCH structure. Although the methyl-lysine recognition is only achieved through the C5HCH domain that appears to fold independently of the PHD, the entire PHDvC5HCH zinc finger cassette is required for effective binding to H3K27me3 (62). Interestingly, the H3K27me3 binding function appears to be unique to NSD2 and diverged from the NSD1 and NSD3 paralogs that also contain a PHDvC5HCH but do not bind to H3K27me3 substrates with similar affinity (62). The discovery that the PHDvC5HCH domain of NSD2 can bind to H3K27me3 *in vitro*, supports a molecular mechanism whereby the methyltransferase activity of NSD2 is recruited to H3K27me3 chromatin regions *via* PHDvC5HCH where it can methylate H3K36 to promote gene transcription and de-repression of chromatin (62).

### Lysine demethylases

The activation of genes that are silenced under facultative heterochromatin coincides with a reduction of H3K27me3. In humans, demethylation of H3K27me3 chromatin is mainly achieved by two proteins: Ubiquitously transcribed tetratricopeptide repeat X chromosome (UTX a.k.a. KDM6A) and Jumonji D3 (JMJD3 a.k.a. KDM6B) (Fig. 1). Both demethylases belong to the Jumonji C (JmjC) domains family of proteins and cooperate with the mixed lineage leukemia (MML) family of H3K4-specific methyl transferases to rapidly stimulate transcription of silenced genes during cellular differentiation and as part of the inflammatory response (63, 64).

**Figure 6:**
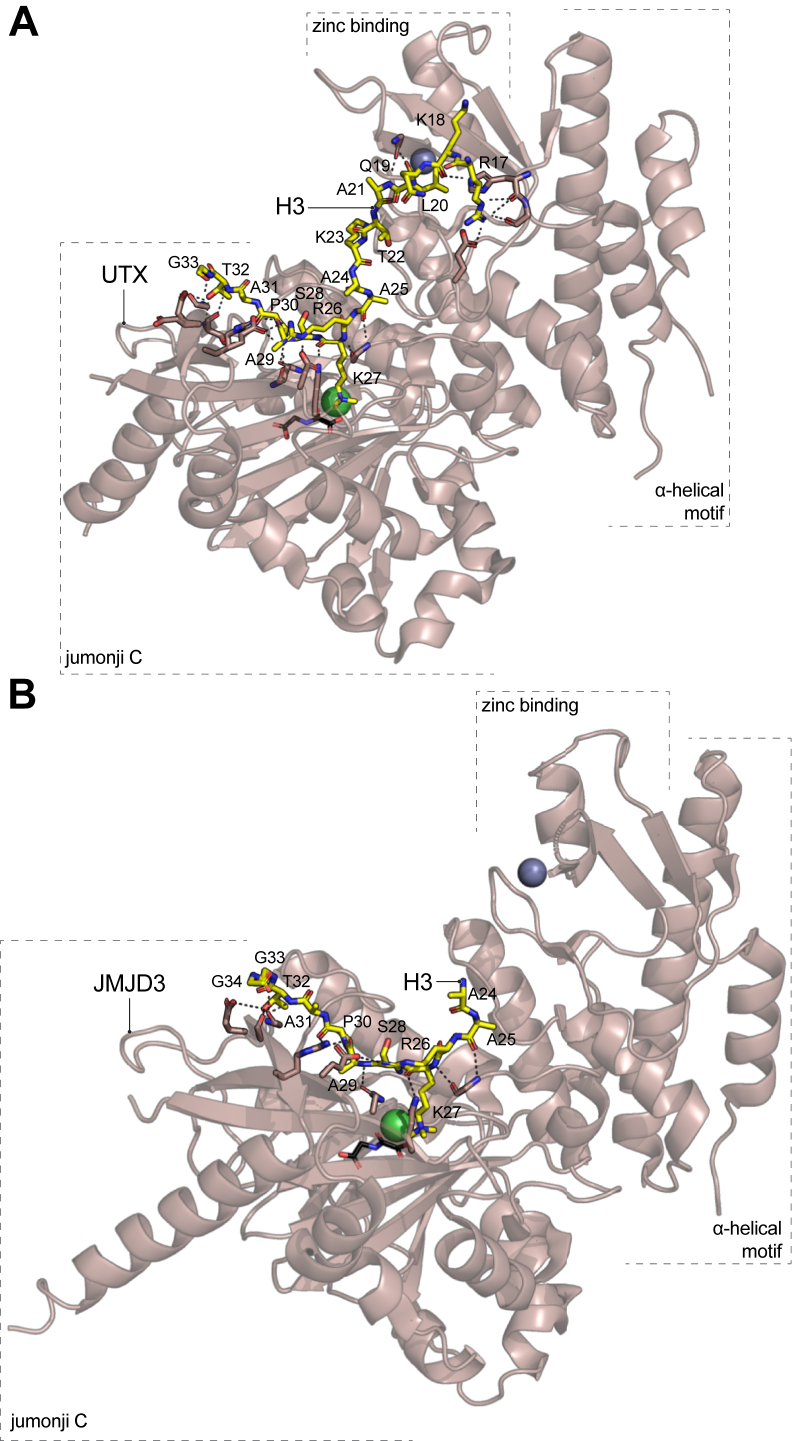
**H3K27-specific demethylases do not rely on aromatic cages for the recognition of the H3K27me3 substrate and make an extensive hydrogen bonding network with H3 residues around K27**. X-ray crystal structures of H3K27me3 peptides in complex with the inactive lysine demethylases *Homo sapiens* UTX (*A*, PDB ID: 3AVR), and *Mus musculus* JMJD3 (*B*, PDB ID: 4EZH). The H3 peptides are represented in a stick model with their amino acids labeled in *black* letters. The demethylases are shown in a cartoon representation with sections of some residues that participate in the binding to the H3 peptide shown in stick models. Hydrogen bonds between the H3 peptide and the demethylases are represented as *dottedlines*. *Silver spheres* represent coordinated Zn^2+^ ions present in the crystal structures. *Green spheres* correspond to Ni^2+^ ions that substituted the functional Fe^2+^ cofactor for crystallization. Shown in a *black* stick model is the N-oxalylglycine (NOG) molecule, a nonreactive analogue of the α-ketoglutarate cofactor, that was cocrystallized with the demethylases. The K27me3 side chain of the H3 peptide fits into the active site cavity of UTX and JMJD3 that is also occupied by NOG and Ni^2+^ in the crystal structures. H3K27me3, Histone H3 tri-methyl lysine 27; JMJD3, Jumonji D3; PDB, Protein Data Bank; UTX, ubiquitously transcribed tetratricopeptide repeat X chromosome.

Although histone demethylases are not chromatin readers in the traditional sense, essential to their function is the initial molecular recognition of their methylated substrate by their catalytic domain. The demethylases UTX and JMJD3 recognize the methylated substrate through a C-terminal segment that is composed of a Jumonji C catalytic domain, a zinc coordinating region, and an alphahelical scaffolding motif (65, 66). In addition, the jumonji C domains of UTX and JMJD3 require Fe^2+^ ions and α-ketoglutarate as cofactors for their enzymatic function. Crystallographic studies of inactive C-terminal regions of human UTX and mouse JMJD3 in complex with an H3K27me3 peptide showed that the jumonji C domain adopts a central β-barrel globular structure that is surrounded by short α-helices and packed against the alphahelical structure and the zinc binding motif (Fig. 6, *A* and *B*) (65, 66).

In UTX and JMJD3, the methylated K27 residue fits into a deep acidic cavity within the main β-barrel structure of the Jumonji domain orienting the trimethyl moiety near the Jumonji cofactors that are located at the bottom of the cavity (Fig. 6, *A* and *B*). Unlike the H3K27me3 reader domains, the cavity that recognizes the methylated lysine is not an aromatic cage and it is mainly formed by polar residues including Asp, Ser, Glu, and only one Phe that together interact with the methylated lysine with a network of CH-O hydrogen bonds (65, 66). The rest of the H3K27me3 peptide mostly interacts with the surface of the Jumonji C domain but can also extend to the zinc-binding domain surface in UTX (Fig. 6*A*). Although several hydrogen bonds and hydrophobic interactions are observed along the H3 peptide and the demethylases, critical substrate contacts that are conserved between UTX and JMJD3 include the recognition of H3R26 that makes hydrogen bonds with Asp and Glu, and H3P30 that makes hydrophobic contacts with another proline ring in the Jumonji domains (Fig. 6, *A* and *B*). Amino acid substitutions in H3R26 and H3P30 or to the demethylase residues that interact with these H3 residues substantially reduced the catalytic activity of the enzyme toward an H3K27me3 substrate, indicating a functional relevance of these sequences in the H3K27me3 recognition (65, 66).

## H3K27me3 binding in a chromatin relevant context

Most of our understanding regarding the molecular recognition of H3K27me3 is in the context of short histone H3 tail peptides. However, although histone tail peptides can be found *in vivo* through the proteolysis of histones (reviewed in (67)), the main biological role of histone modifications is thought to be in the context of the nucleosome. Notably, in this context the histone tails are not always found solvent exposed, but rather are found to form robust, though transient and heterogenous interactions with nucleosome surfaces such as nucleosomal DNA and other histone tails (68, 69, 70, 71, 72, 73). These nonspecific interactions can have a strong regulatory effect on histone PTM read-out and the investigation of histone PTM recognition in the context of the nucleosome is an important emerging field of scientific research.

**Figure 7:**
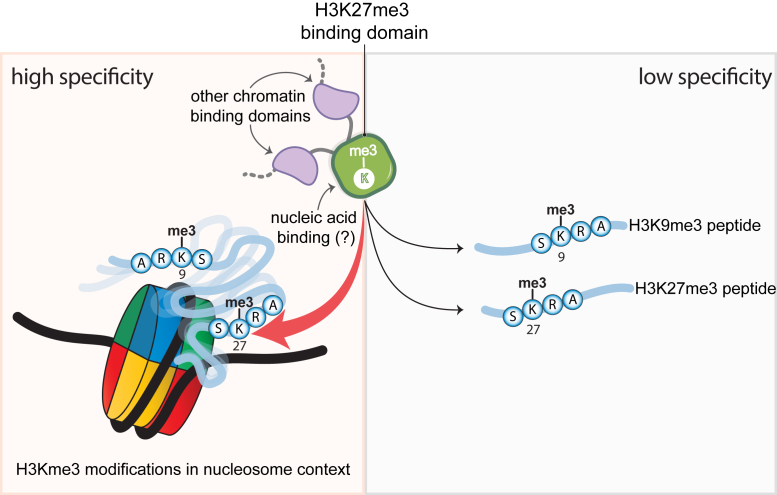
**The nucleosome can refine the selectivity of H3K27me3 readers**. *In vitro*, the chromodomain of CBX7 does not discriminate between peptides of H3K9me3 and H3K27me3. However, when the marks are part of the entire nucleosome, the CBX7 chromodomain exclusively binds to the H3K27me3-modified nucleosomes, indicating that the nucleosome structure can regulate histone PTM read-out. Using a different protein region than the H3K27me3 binding site, the chromodomain of CBX8 can also interact with DNA. Therefore, synergistically the histone and DNA functions of the CBX8 chromodomain can presumably influence histone PTM preference in the context of the nucleosome. Similarly, functional domains neighboring the H3K27me3 reader domains, like the AT-hooks that can bind to DNA and are adjacent to the chromodomains in CBX proteins, are likely to also affect affinity toward specific nucleosome substrates. These constitute examples of the emerging nucleosome regulation model where the chromatin context controls accessibility to H3K27me3. CBX, chromobox; H3K27me3, Histone H3 tri-methyl lysine 27; PTM, posttranslational modification.

In the case of H3K27me3, recent evidence suggests that the nucleosome context is quite important. It was recently found that the CBX7 chromodomain cannot discriminate between histone tail peptides containing either H3K27me3 or H3K9me3 but rather binds both peptides with similar affinities *in vitro* (11). However, in the context of the nucleosome, the CBX7 chromodomain exhibited a strong preference for H3K27me3 over H3K9me3, indicating that the nucleosome structure can “fine tune” histone specificity (11) (Fig. 7).

Similarly, some H3K27me3 reader domains contain interacting surfaces outside of the methyl-lysine binding site that can engage with the nucleosome and perhaps favor selectivity for H3K27me3. In the CBX paralogues, the chromodomain of CBX8 has been demonstrated to have a basic patch that binds to DNA in a nonsequence specific manner including nucleosomal DNA (74). It has been proposed that both the DNA and H3K27me3 binding functions of the chromodomains of CBX8 can act synergistically to engage with H3K27me3 nucleosomes and regulate gene expression (74) (Fig. 7)

Finally, there is evidence that reader recognition of histone PTMs in the chromatin context is likely to be facilitated or enhanced by adjacent domains within the same polypeptide or additional domains in other subunits of a macromolecular complex (reviewed in (75)). For instance, besides the H3K27me3-binding chromodomains, the CBX2/4/6/7/8 paralogues contain AT-hook domains and intrinsically disorder regions that can bind to nucleosomal and linker DNA (11, 76, 77, 78, 79). Therefore, H3K27me3 chromatin recognition by CBX proteins is likely to be a result of multiple low affinity binding events that positions the chromodomain near H3K27me3 within the nucleosome (Fig. 7).

## Pharmacological targeting of the H3K27me3 axis

**Figure 8:**
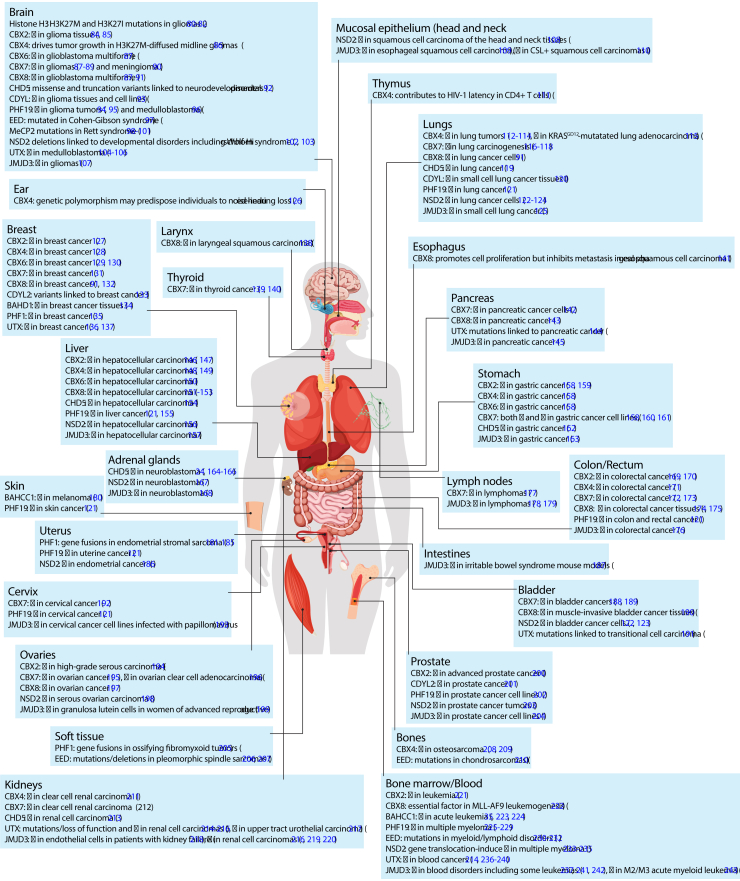
**Proteins that participate in the H3K27me3 axis are implicated in several human diseases**. The misregulation of H3K27me3-related proteins can include protein overexpression (↑ - oncogenic role) or underexpression (↓ - tumor suppression role), as well as amino acid substitutions, deletions or protein fusions that alter the native behavior of the protein and contribute to disease phenotypes. Remarkably, the brain and the nervous system are the most impacted organs by the misregulation of H3K27me3-related proteins followed by the bone marrow/blood, breast, lungs, colon/rectum, liver, and stomach. H3K27me3, Histone H3 tri-methyl lysine 27.

The dynamic regulation of facultative heterochromatin operates as a gate for gene expression and H3K27me3 is the pivotal “axis” that blocks or permits gene expression profiles that confer cell lineage identity. As discussed above, the function of this H3K27me3 axis is centrally regulated by the catalytic activity of PRC2, can be actively counterbalanced by UTX and JMJD3 demethylases, and is recognized by many reader domains to signal specific functions at H3K27me3 loci. Giving the relevance of H3K27me3 in cell identity, it is not surprising that all the proteins involved in H3K27me3 regulation or function are implicated in human diseases with cancer being the most well studied (Fig. 8).

Intriguingly, a vast majority of H3K27me3-related proteins are found misregulated in the brain and nervous system including the histone H3 itself where the missense mutations K27M and K27I that prevent K27 methylation by PRC2 have been mapped to gliomas frequently found in pediatric patients (80, 81, 82) (Fig. 8). Other organs that show a prevalent number of H3K72me3-proteins misregulated in human malignancies are the lungs, stomach, breast, liver, colon/rectum, and bone marrow (Fig. 8). Surprisingly, despite the functional relevance of the PRC2 subunit EED in the recognition and spreading of H3K27me3, reports indicating altered functions of the EED subunit are scarce, with a handful of studies indicating misfunction in the Cohen-Gibson syndrome, blood disorders, and in bone and soft tissue sarcomas (Fig. 8). This is in stark contrast to the PRC2 catalytic subunit EZH2 that has been reported in several malignancies including multiple cancers (reviewed in (83)).

Efforts to pharmacologically disrupt H3K27me3-protein interfaces in disease contexts have been largely focused on the development of small molecule inhibitors that preferentially bind to the methylated lysine binding pocket of the protein, therefore blocking the recognition of the native H3K27me3 chromatin. However, some strategies also include the design of competitor peptides, allosteric modulators, and protein degrading molecules. Applicability of these drugs is mostly suitable to cases where the target protein is found upregulated, which is the trend for H3K27me3-related proteins in several cancers (Fig. 8).

### Inhibitors of PRC2

**Figure 9:**
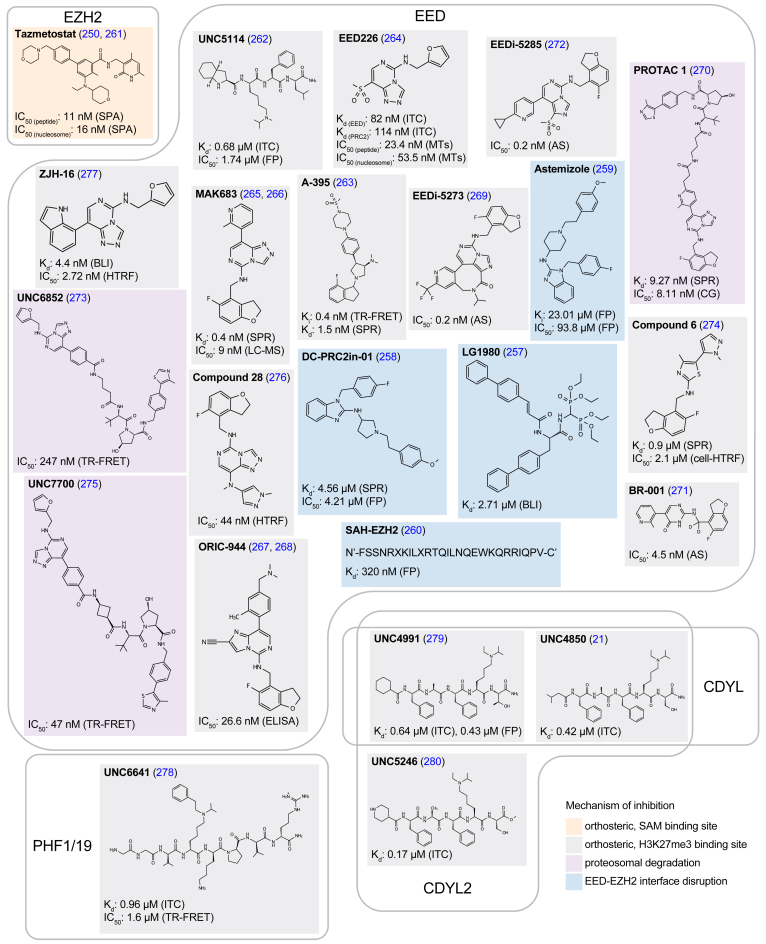
**Chemical structure of small molecule compounds developed to inhibit the function of PRC2 proteins**. Besides developing molecules that inhibit the catalytic function of EZH2, several compounds have been engineered to target the EED subunit that recognizes H3K27me3. These compounds can bind to the aromatic cage in EED, disrupt the interaction between EZH2 and EED or induce the proteosome degradation of the entire PRC2 complex. PHF1/19 and CDYL/2 also associate with PRC2 and molecules that inhibit the recognition of H3K27me3 by the Tudor domain in PHF1/19 and the chromodomain in CDYL/2 have also been developed. AS, AlphaScreen assay; BLI, bio-layer interferometry; cell-HTRF, cell-based homogenous time-resolved fluorescence; EED, embryonic ectoderm development; ELISA, enzyme-linked immunosorbent assay; FP, fluorescence polarization; H3K27me3, Histone H3 tri-methyl lysine 27; HTRF, homogenous time-resolved fluorescence; ITC, isothermal colorimetric titration; LC-MS, liquid chromatography-mass spectrometry; MT, methyltransferase assay; PRC2, polycomb repressive complex 2; SPA, scintillation proximity assay; SPR, surface plasmon resonance; TR-FRET, time-resolved fluorescence resonance energy transfer.

In many cancers, H3K27me3 can abnormally accumulate at gene promoters silencing tumor suppressor genes and driving progression of the disease (244). Since PRC2 is the only known factor that can establish H3K27me3, a reasonable strategy to mitigate H3K27me3-dpendent diseases has been the development of drugs that disrupt the catalytic activity of PRC2. The earliest efforts on this quest focused specifically on developing small molecules that can bind to the EZH2 subunit to directly impair the methyltransferase activity of PRC2 (245), followed by efforts to develop these inhibitors into protein degraders (246, 247, 248). These are reviewed in (249). Currently, the best-studied inhibitor of EZH2 is Tazemetostat (Fig. 9), a small molecule that selectively binds to the cofactor SAM binding pocket in EZH2 (Fig. 5) competing off endogenous SAM binding and preventing methylation of the histone H3 (250). Because of its mechanism of action and specificity to EZH2, Tazemetostat is a first-in-class drug that received approval from the United States Food and Drug Administration (FDA) in 2020 for the treatment of epithelioid sarcoma and some lymphomas, where EZH2 can also be found mutated and/or upregulated (251).

However, in many cases the enzymatic inhibition of EZH2 does not phenocopy the anticancer effects of the genetic deletion, possibly due to nonenzymatic functions of PRC2 (252). Although this may be mitigated by EZH2 protein degraders (253), some cancers can develop resistance to EZH2 inhibitors such as Tazemetostat, through the upregulation of EZH1 or through mutation of EZH2 (254, 255, 256). To combat these issues, there have been efforts to inhibit PRC2 function by targeting other subunits besides EZH2, including the EED protein that recognizes H3K27me3 (Fig. 5). Although strategies to disrupt the interaction between EZH2 and EED have been explored as a mechanism of PRC2 inhibition, such as DC-PRC2in-01, LG1980, Astemizole, and SAH-EZH2 (257, 258, 259, 260) (Fig. 9), the vast majority of the efforts in the development of drugs that target the EED subunit has been focused on engineering molecules that bind directly to the aromatic cage of the EED protein to block H3K27me3 recognition (Fig. 9).

UNC5114 (Fig. 9), binds to the H3K27me3 binding pocket of EED further preventing SRM helix stabilization (Fig. 5) and subsequent PRC2 enzymatic activation (262). A-395 and EED226 (Fig. 9) bind EED through an induced fit mechanism that produces a substantial conformational rearrangement of the EED residues in the aromatic cage (Y365, Y148, and F97. Fig. 5) resulting in the inhibition of both basal and H3K27me3-stimulated PRC2 catalytic activities (263, 264, 281, 282). MAK683 (Fig. 9) has gone through PhaseI/II clinical trials and is found to be efficacious and well tolerated (265, 283, 284). Similarly, ORIC-944 and EEDi-5273 (a.k.a. APG-5918) are in clinical trials for prostate cancer (267) and anemia/lymphomas (285, 286, 287), respectively. Finally, bivalent chemical degraders, or proteolysis targeted chimeras (PROTAC), are emerging as an attractive strategy to induce the degradation of the PRC2 complex in the clinic (270, 273, 275, 288). The bivalent degraders UNC6852 and UNC770 are based on EED226 while PROTAC 1 uses MAK683 as the moieties that bind to the EED subunit (Fig. 9). In all three structures, however, the rest of the compound induces degradation of PRC2 through the recruitment of the VHL ubiquitin ligase (270, 273, 275).

Other proteins that regulate PRC2 activity and that have been considered as therapeutic targets include PHF1/19. Specifically, PHF1/19 can physically associate with PRC2 to form substoichiometric variants of the complex (6, 43, 47, 289) and are implicated in many diseases. PHF19 is often found upregulated while PHF1 exhibits gene fusions and deletions (Fig. 8). Drug development to target the histone H3 reader function of the Tudor domain in PHF1/19 is slowly emerging with the small molecule UNCC6641 being one of the pioneers in the field (278) (Fig. 9). Although UNCC6641 was intended to inhibit the recognition of the H3K36me3 mark by the PHF1/19 Tudor domains, it is likely to prevent binding to H3K27me3 substrates as well. Despite some modifications for improving pharmacokinetics, the compound still has low cell permeability, currently limiting the ability to assess how the inhibition of the PHF1/19 Tudor domains affects overall gene repression by PRC2 (278).

Similarly, the specific recognition of H3K27me3 substrates by the chromodomains of CDYL/2 has also been linked to PRC2 activity (21, 23); in addition to having a critical role in neuronal development and the overlapping recognition of the H3K9me2/3 marks (21, 56, 290, 291, 292, 293). Inhibitors that block histone H3 binding of the chromodomains in CDYL/2 have been developed (21, 279, 280) (Fig. 9). However, few reports link misregulation of CDYL/2 proteins to human diseases (Fig. 8), therefore they are not strongly considered as therapeutic targets and there are limited efforts to improve CDYL inhibitors for cellular studies.

### Inhibitors of CBX chromodomains (PRC1)

**Figure 10:**
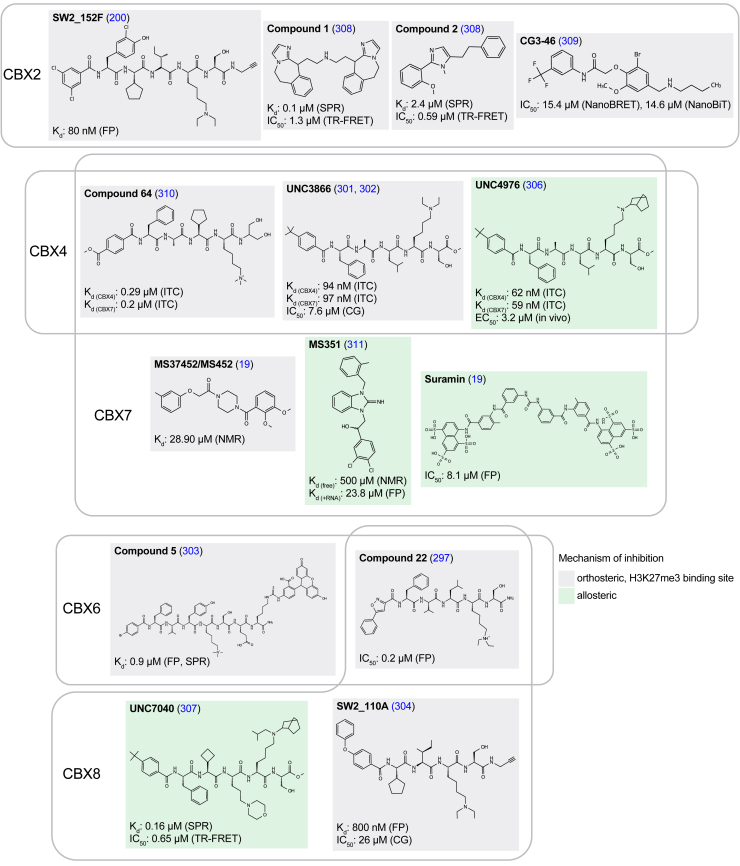
**H3K27me3 inhibitors of the PRC1-CBX chromodomains are generally characterized by a peptidic nature with overlapped specificity between paralogues**. Due to the high degree of sequence and structure similarity between the chromodomains of the CBX2/4/6/7/8 proteins the development of small molecule inhibitors that prevent the recognition of H3K27me3 have often yielded overlapping specificity between the paralogues. In addition, the implementation of peptide-based drug design has been most successful at targeting the large and shallow binding pockets in these chromodomains. CBX, chromobox; CG, cell growth assay; FP, fluorescence polarization; H3K27me3, Histone H3 tri-methyl lysine 27; ITC, isothermal colorimetric titration; NanoBiT, NanoLuc Binary Technology; NanoBRET, NanoLuciferase Bioluminescence Resonance Energy Transfer; SPR, surface plasmon resonance; TR-FRET, time-resolved fluorescence resonance energy transfer.

Synergistic to PRC2 function, gene repression can also be controlled by PRC1, which catalyzes the enzymatic deposition of the H2AK119Ub mark in nucleosomes and physically compacts chromatin (294) (Fig. 1). The CBX proteins form mutually exclusive complexes of PRC1 and the recognition of H3K27me3 by the chromodomains in the CBX proteins is central to the functional crosstalk between PRC1 and PRC2, with evidence suggesting that the CBX paralogues play specific and essential roles during development (reviewed in (295)). Consequently, CBX proteins are implicated in numerous diseases with CBX2/4/8 frequently exhibiting oncogenic properties while CBX6/7 predominantly having tumor suppressor roles (Fig. 8). Blocking the recognition of H3K27me3 by the chromodomains in the CBX proteins is an attractive pharmacotherapy venture that in some cases could promise lower toxicity and greater specificity than directly inhibiting the catalytic function of the PRC1/2 complexes (296). However, while the CBX paralogues substantially differ in sequence and domain composition outside of the chromodomains, the high degree of homology between the CBX chromodomains make the development of inhibitors selective for specific CBX paralogues challenging (297, 298) (Fig. 10). In addition, CBX chromodomains bind to H3K27me3 through an induced fit mechanism with low binding affinity (200–700 μM) (18, 74, 299) and employing large and shallow binding pockets (Fig. 2, *B*–*D*), making the development of small molecule inhibitors difficult. Nevertheless, computational analysis of the CBX chromodomains structures suggests that these domains can be considered druggable, exhibiting similar potential than other methyl lysine binding readers (300).

The first breakthrough in the development of CBX chromodomain inhibitors came from the discovery that these domains can also bind to peptides of the SETDB1 protein that were trimethylated at the residue 117, exhibiting a 100-fold increase in affinity when compared to H3K27me3 peptides (18). The use of a SETDB1 peptide as a starting point resulted in the development of UNC3866, a potent inhibitor of CBX4/7 (301, 302) (Fig. 10). Peptides selective for CBX6, compound 5 and 22, have been identified from small solution-phase libraries (297, 303); while some peptidic compounds selective for CBX8, SW2_110A, and CBX2, SW2_152F, were identified from focused DNA-encoded libraries (200, 304, 305) (Fig. 10). Interestingly, the chromodomain inhibitors UNC4976 and UNC7040 for CBX7 and CBX8 (Fig. 10), respectively, were discovered as cellular positive allosteric modulators (PAM) that bind to the H3K27me3 binding pocket and simultaneously alter the conformation of the aromatic cage to favor nucleic acid binding (306, 307).

Currently, despite the progress in the development of molecules that block the recognition of H3K27me3 by the CBX chromodomains *in vitro*, one of the major limitations for therapeutic applicability of the CBX inhibitors is the low cell permeability due to the presence of charged amines and the peptidic nature of many of these compounds (Fig. 10). Efforts to overcome this challenge have included the incorporation of norbornane derivates as well as appending cyclic amines in CBX7/8 inhibitors to increase potency (306, 307, 312). Efforts to identify nonpeptidic inhibitors have resulted in several small molecule inhibitors of CBX7 and CBX2: compound 1, compound 2, CG3-46, MS37452, and MS351 (Fig. 10) (19, 308, 309, 311). However, the identified small molecule inhibitors bind CBX chromodomains with either low affinity or no demonstrated selectivity with submicromolar affinity, limiting their utility.

### Inhibitors of nonpolycomb proteins

**Figure 11:**
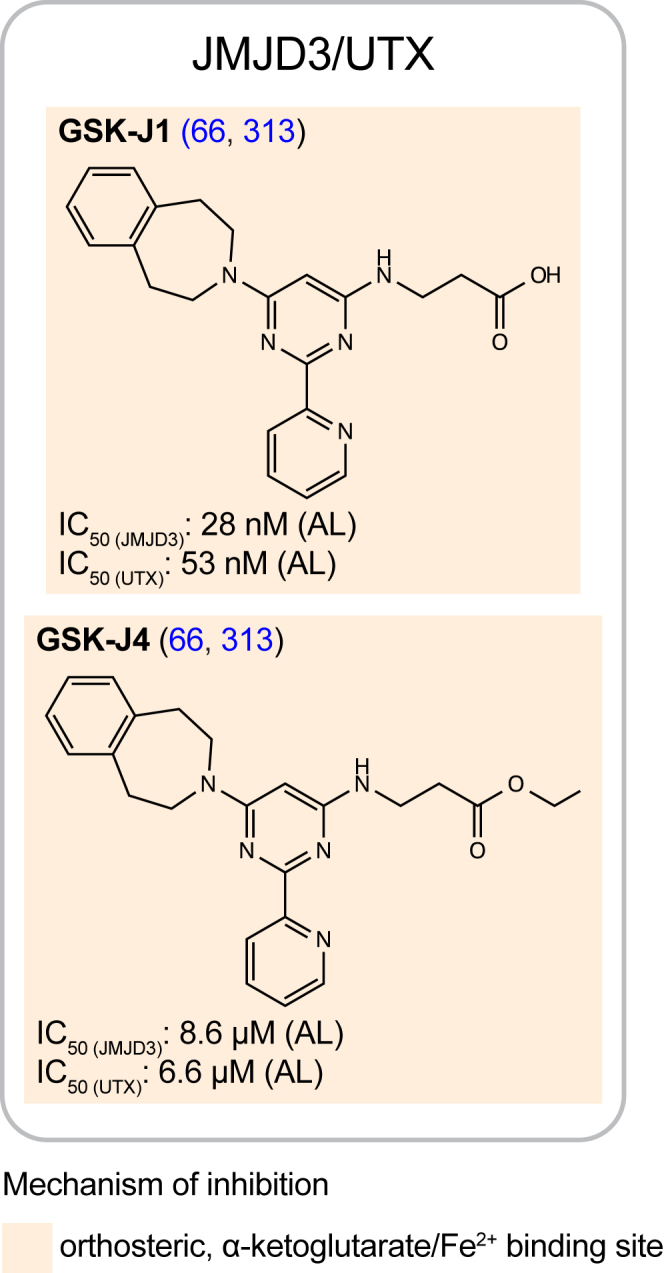
**GSK-J1 and its prodrug derivate, GSK-J4, are the most potent inhibitors of the UTX/JMJD3 H3K27me3 demethylases**. GSK-J1 competes off the cofactor α-ketoglutarate and mimics P30 of the histone H3 peptide to prevent the demethylation of H3K27me3 chromatin by UTX and JMJD3. GSK-J4 has enhanced cell permeability compared to GSK-J1 and its metabolization inside the cell yields the pharmacologically activate GSK-J1 form. AL, AlphaLISA assay; H3K27me3, Histone H3 tri-methyl lysine 27; JMJD3, Jumonji D3; UTX, ubiquitously transcribed tetratricopeptide repeat X chromosome.

As mentioned above, the lysine demethylases UTX and JMJD3 (KDM6A/B) both recognize and demethylate H3K27me3 nucleosomes. The most prominent inhibitor for these demethylases, GSK-J1 (Fig. 11), was discovered from screening the nearly two million compounds of the GlaxoSmithKline corporate compound collection; it inhibits both JMJD3 and UTX, with an IC_50_ of 28 and 53 nM, respectively (66, 313). The small molecule GSK-J1 has a carboxylic acid that mimics α-ketoglutarate along with an aromatic ring that mimics P30 in the histone H3 peptide (66) (Fig. 6). Structurally, however, the binding is more complicated than a simple bivalent inhibitor mechanism as GSK-J1 binding to UTX/JMJD3 results in a shift in the position of the divalent cation such that GSK-J1 acts competitively with α-ketoglutarate and noncompetitively with H3K27me3 (66). To facilitate cell permeability, the carboxylic acid in GSK-J1 was converted to the ester derivate in a prodrug strategy that resulted in the GSK-J4 compound (66) (Fig. 11). GSK-J4 has been used extensively in preclinical research as an anti-inflammatory and in cancer and infectious diseases models, mostly in cases where JMJD3 is found misregulated (reviewed in (314)) (Fig. 8); however, it has limited selectivity for UTX/JMJD3 over KDM5 demethylases, limiting its utility (313).

For the other reader domains: CHD5, BAHCC1, BAHD1, NSD2, and MeCP2, despite being implicated in human malignances (Fig. 8), inhibitors to target their H3K27me3 binding function have not yet been developed. NSD2, however, is an active target for pharmacological intervention with extensive drug development aiming to regulate its H3K36me2-realted functions by inhibiting the catalytic SET and reader PWWP1 domains (reviewed in (315)). As research continues to expand our understanding of the biological role of these and emerging H3K27me3 readers, efforts to develop compounds to disrupt relevant interfaces will likely follow.

## Conclusions and outlook

The recognition of H3K27me3 in biological systems is functionally and structurally diverse. The proteins that specifically recognize H3K27me3 within heterochromatin (Table 1) evolved to adapt distinct molecular mechanisms of recognition beyond just the Kme3 moiety, with chromodomains and Tudor domains mainly engaging the H3K27me3 substrate through residues upstream of K27 (Figs. 2 and 4), while H3P30 exclusively plays a critical role in binding to BAH and H3K27me3 mammalian demethylases (Figs. 3 and 6). Interestingly, the seemingly minimal level of interaction between PRC2/EED and H3K27me3 (Fig. 5) may favor the spreading of H3K27me3 onto neighboring unmodified nucleosomes by preventing the anchoring of PRC2 to a fixed genomic locus.

Importantly, interactions beyond the methyl-lysine modification are fundamentally important to the appropriate functional recognition of H3K27me3 regions as residues around H3K27 can be found modified with other PTMs (Fig. 1) further modulating affinity to K27me3 and adding an additional layer of regulation. Furthermore, future high-resolution structural determination of novel H3K27me3 binding proteins in complex with the H3 substrate, such as MeCP2, will continue to expand our knowledge on the regulatory versatility of the H3K27me3 facultative heterochromatin.

Although, the implementation of histone tail peptides has permitted the structural characterization of protein–histone tail interactions at a high-resolution level, in chromatin the histone tails are not always readily accessible and are in involved in nonspecific interactions with nucleosomal surfaces, such as DNA, posing a regulatory mechanism for histone PTM read-out, like in the case of the chromodomain of CBX7 (Fig. 7). However, the specific details of how the nucleosome structure grants histone PTM accessibility to specific factors are slowly emerging. Questions that remain currently unanswered include the following: How does the presence of the H3K27me3 modification in the nucleosomes affect the dynamics and accessibility of the H3 residues at and around K27? What are the requirements for factors to bind H3K27me3 in the context of the nucleosome? How is specificity in the nucleosome context achieved? Addressing these questions will substantially expand our understanding of the H3K27me3 heterochromatin function. As more H3K27me3-related factors continue to be investigated in the presence of chromatin-derived H3K27me3 substrates, the molecular mechanism that specifically regulate H3K27me3 read-out are likely to be elucidated and importantly will further inform therapeutic efforts.

Efforts to expand our understanding of H3K27me3 function in human health and disease will continue to illuminate targets for pharmacological intervention. Conspicuously, most of the H3K27me3-related proteins, including the histone H3, are implicated in diseases that particularly affect the brain and nervous system (Fig. 8). In addition, giving the essential role of the H3K27me3 facultative heterochromatin in cellular identity, it is not surprising that the misregulation of the factors that recognize H3K27me3 coincides with cancer phenotypes (Fig. 8).

The design of inhibitors that specifically hamper H3K27me3 recognition by chromatin factors substantially vary in chemical nature, with a marked tendency for peptide-base molecules in the case of the chromodomains from CDYL and CBX proteins, and Tudor domains in PHF1/19 (Figure 9, Figure 10, Figure 11). In addition, the mechanisms of inhibition are not only limited to compounds that directly bind to the H3K27me3-binding pocket, but in the case of PRC2, also extends to inhibitors that disrupt the complex formation and compounds that promote degradation (Fig. 9).

Giving the current developments on the understanding of the dynamic nature of the nucleosome, that histone tails are found in a heterogenous conformational ensembles within the chromatin context, and that the nucleosome structure can serve as a regulatory factor of histone PTM read-out, it is likely that these characteristics will soon begin to be incorporated as part of pharmacological intervention to treat H3K27me3-related diseases.

## Conflict of interest

The authors declare that they have no conflicts of interests with the contents of this article.
